# Prognostic value of long non-coding RNA BLACAT1 in patients with papillary thyroid carcinoma

**DOI:** 10.1186/s12935-018-0544-9

**Published:** 2018-03-27

**Authors:** Denghui Liao, Gang Lv, Ting Wang, Jie Min, Yadong Wang, Shengchun Liu

**Affiliations:** 1 Department of Endocrinology and Breast Surgery, the First Affiliated Hospital of Chongqing Medical University, Chongqing, 400043 China; 2 Department of Breast and Thyroid, Chongqing Traditional Chinese Medicine Hospital, Chongqing, 400021 China

**Keywords:** Long noncoding RNAs (lncRNAs), Papillary thyroid cancer (PTC), Metastasis, Survival

## Abstract

**Background:**

Long noncoding RNAs (lncRNAs) have been reported to have potential diagnostic and prognostic values for numerous cancers. However, the plasma expression of lncRNA BLACAT1 and its clinical value in patients with papillary thyroid cancer (PTC) remain unknown.

**Methods:**

The expression profile of BLACAT1 in 87 PTC patients (case group) and 36 patients with nodular goiter (control group) were investigated by qRT-PCR. The Kaplan–Meier method was used for RFS curves, and the univariate survival differences were analyzed by the log-rank test.

**Results:**

BLACAT1 expression was downregulated in the plasma of case group compared with control group (P = 0.003). We also found that low plasma BLACAT1 expression was correlated with lymph node metastasis (LNM) (P < 0.001). Multivariate analysis showed that BLACAT1 was an independent risk factor for lymph node metastasis and gender (P < 0.05). The area under the ROC curve for BLACAT1 was 0.825 in LNM prediction (P < 0.001).

**Conclusion:**

The present study demonstrated that BLACAT1 could act as a possible suppressor gene in PTC and may serve as a potential biomarker for prognosis prediction of PTC.

## Background

Thyroid carcinoma is the most common endocrine neoplasm worldwide [1]. About 300,000 patients are diagnosed with thyroid carcinoma each year. The median age at the time of diagnosis is 50 years old, and approximately 40,000 people die from thyroid carcinoma. Papillary thyroid carcinoma (PTC) is the most prevalent type of thyroid carcinoma, accounting for 80% of all thyroid carcinomas [2, 3]. Generally, thyroid carcinomas develop slowly and can be cured by thyroidectomy or radioiodine, even for the thyroid metastatic carcinoma [4]. Most patients with PTC have a good prognosis, but a small fraction of patients develop PTC with dedifferentiation, resulting in aggressive features and poor prognosis [5]. At present, newly-found target drug combined treatment changes the effectiveness of thyroid cancer with a poor response to radioiodine [6]. BRAF V600E mutation can affect iodine metabolism and decrease the absorption of ^131^I of the thyroid gland through BRAF/MEK/MAPK pathway. Therefore it may become a potential therapy target for PTC [7]. Researchers have found the relationship between some molecules and invasion in thyroid carcinoma currently [8]. However, new biomarkers in blood for predicting the invasiveness of PTC need to be further identified.

Long non-coding RNAs (lncRNAs) are conservative, non-coding RNAs with more than 200 nucleotides in length. Many lncRNAs have biological functions, such as regulating the transcription and translation of protein-coding sequences [9]. LncRNA can regulate the growth and metastasis of tumors by altering the expression of different molecules and affecting chromatin structure, transcriptional activity, mRNA stability, and transcription process or translation of mRNA [10]. Over the past 10 years, more and more evidence has shown that lncRNAs play an important role in tumor proliferation and metastasis. For example, lncRNA GAPLINC stimulated SNAI2 as a transcriptional vector combined with PSF and NONO to promote the invasion of colorectal cancer [11]. In thyroid carcinoma tissues, Kim et al. found that 56 lncRNAs were related to thyroid carcinoma, and proved that lymph node metastasis (LNM) of thyroid cancer was closely associated with BRAF V600E mutation and LOC100507661 [12]. BLACAT1, also known as linc-UBC1, locus on human chromosome 1q32.1, has been reported to be upregulated and serve as a negative prognostic factor in many types of cancers [13]. However, the clinical and biological significances of BLACAT1 in PTC are still unclear. The purpose of this study was to evaluate the diagnostic and predicting value of BLACAT1 related to survival and recurrence of PTC patients.

## Materials and methods

### Patients and samples

A total of 87 cases of PTC enrolled in Chongqing Medical University from January 2011 to June 2013 and 36 patients with nodular goiter (NG) were included in this study. Patients with other tumors, or tumor history, or received chemotherapy and radiotherapy were excluded. 10 ml fasting blood samples of all patients before surgery were collected. Blood samples were placed in the K2-EDTA tubes, and centrifuged at 800×*g* for 15 min. Plasma samples were immediately transferred to tubes without ribonuclease/deoxyribonuclease. Then phosphate buffered saline was added to 20 μl plasma samples with the ratio of 1:4 for spectrophotometric analysis. The samples were with hemolysis and stored at − 80 °C before use. The diagnosis of PTC or nodular goiter was performed by two pathologists independently. The study was approved by the Clinical Research Ethics Committee of Chongqing Medical University and in accordance with the ethical standards formulated in the Helsinki Declaration and the national regulations. All patients signed informed consent.

### qRT-PCR

Total RNA was isolated from plasma samples with Trizol reagent (Invitrogen Life Technologies Co, Carlsbad, CA, USA) according to the manufacturer’s instruction. The quality of RNA samples was evaluated by ultraviolet spectrophotometer (Bio-Rad, Hercules, CA, USA) and the 260/280 nm absorbance ratio of the RNA samples was at 1.8–2.0. Total RNA (500 ng) was then reverse-transcribed into cDNA with the first strand cDNA synthesis kit (Takara, Tokyo, Japan). Quantitative real-time PCR (qRT-PCR) was performed using ViiA 7 real-time PCR System (Applied Biosystems, Foster City, CA, USA). The primer sequences of lncRNA BLACAT1 and β-actin were designed and synthesized by KangChen Bio-tech (Shanghai, China).

The primer sequences for lncRNA BLACAT1: F 5′-GACAAAGCACAAGCGAAACAAG-3′ and R 5′-GGACATCTGATAGCCTGGTGAC-3′, β-actin: F 5′-GCGACTTTTGGCGAGGATTG-3′ and R 5′-CCTTCCAGTAACACGAATCTATT-3′. PCR reaction was as follows: pre-denaturing 95 °C 15 min, denaturing 95 °C 15 s, 55 °C 45 s, 95 °C 10 s, 35 cycles. The gene expression of all the samples was analyzed and the difference between patients and healthy controls was calculated by 2^−ΔΔCt^. The experiment was repeated three times. Plasma BLACAT1 high expression and BLACAT1 low expression were divided by the median value.

### Statistical analysis

All statistical analyses were performed with SPSS software (version 21.0, Inc., Chicago, IL, USA). The data were expressed as mean and standard deviation. Comparison between groups was performed using Student t-test or non-paired Mann–Whitney test. The qualitative data were expressed as the number of cases or percentages and assessed by χ^2^ test. Receiver operating characteristic curves (ROC) were used to assess the value of BLACAT1 to predict lymph node canceration and LNM. Multivariable logistic regression analysis was used and data were expressed with odds ratio (OR) and 95% confidence interval (95% CI). *P *< 0.05 was regarded as statistically significant.

## Results

### Down-regulation of lncRNA BLACAT1 expression in plasma of PTC patients

**Fig. 1:**
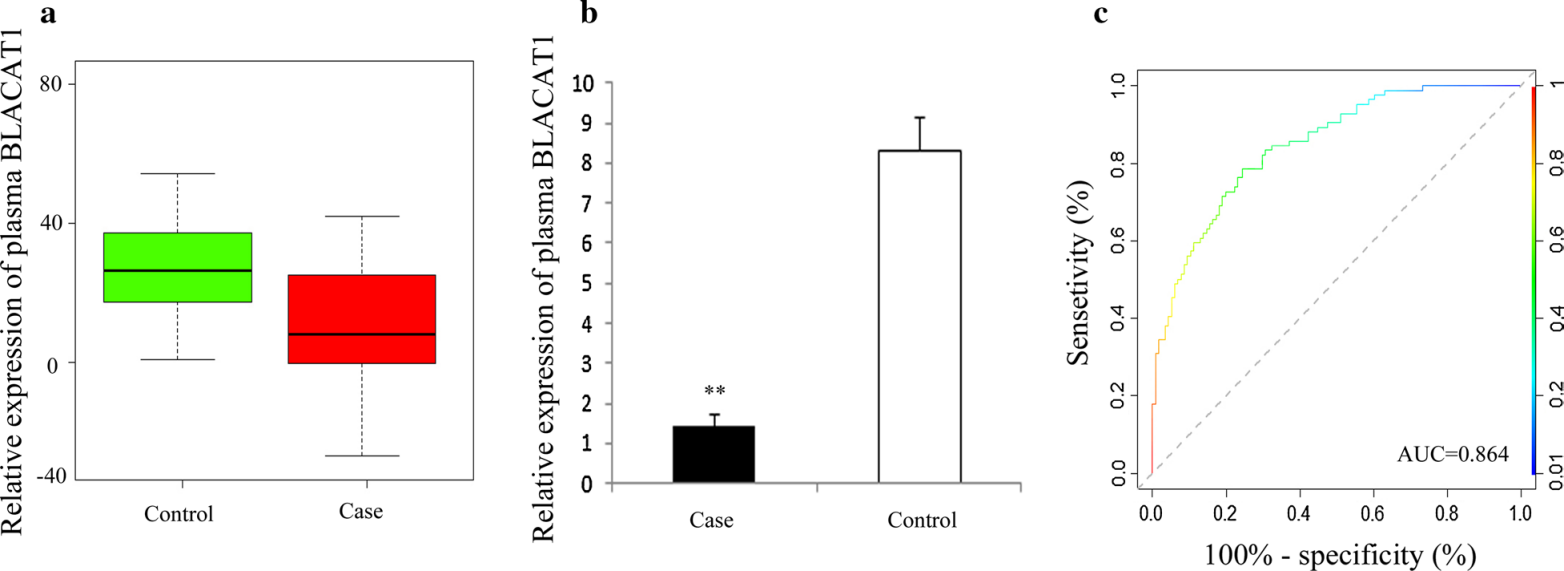
RT-qRCR and ROC curve analysis of BLACAT1 as a diagnostic marker for PTC. Box plot (**a**) and histogram (**b**) of expression of BLACAT1 in plasma of patients with PTC (n = 87) and nodular goiter (n = 36); **c** ROC curve for evaluating the diagnostic value of BLACAT1 (*P* < 0.001)

As compared with the control group, the expression of BLACAT1 in plasma was downregulated in PTC patients (P = 0.003). Multivariable analysis showed that down-regulation of BLACAT1 in plasma was an independent risk factor for PTC (Table 1). The diagnostic value of BLACAT1 on nodular goiter and PTC was evaluated by ROC curve. The AUC was 0.864 (95% CI 0.634–0.821, P < 0.001). The sensitivity and specificity were 89.53 and 86.91%, respectively (Fig. 1).

**Table 1:** Association between lymph node metastasis and lncRNA BLACAT1 expression by multivariate analysis

	lncRNABLACAT1		
	OR	95% CI	P value
PTC diagnosis	0.887	0.833–0.982	0.013*
Model 1			
Lymph node metastasis			
Model 1	0.596	0.459–0.805	< 0.001**
Model 2	0.504	0.341–0.798	0.005*

Model 1, adjusted for sex and age. Model 2, adjusted for sex, age, thyroid-stimulating hormone pre-operation, tumor size, extra thyroidal extension, multifocality, nodular goiter, and Hashimoto thyroiditis

* P < 0 05 and ** P < 0.001

### Correlation between lncRNA BLACAT1 and clinicopathological traits

To explore the correlation between plasma lncRNA BLACAT1 and clinicopathological traits, PTC patients were assigned into low and high expression groups based on the median expression value of BLACAT1. Our data demonstrated that down-regulation of lncRNA BLACAT1 expression was associated with LNM and gender (*P *< 0.05). However, no correlation was found between lncRNA BLACAT1 and extrathyroidal extension, age, tumor size, or advanced TNM stage (Table 2).

**Table 2:** Association between lncRNA BLACAT1 expression and clinicopathological traits in PTC patients

Parameters	lncRNA BLACAT1		χ^2^	P
	Low (%)	High (%)		
	(n = 44)	(n = 43)		
Gender				
Male	15 (34.09)	7 (16.28)	5.122	0.029*
Female	29 (65.91)	36 (83.72)		
Age (years)				
< 45	20 (46.94)	25 (58.14)	1.825	0.317
≥ 45	24 (53.06)	18 (41.86)		
Extrathyroidal extension				
Positive	24 (54.55)	20 (46.51)	0.716	0.581
Negative	20 (45.45)	23 (53.49)		
Tumor size (cm)				
≤ 1	28 (63.64)	32 (74.42)	1.105	0.426
> 1	16 (36.36)	11 (25.58)		
TNM staging				
I/II	32 (72.73)	36 (83.72)	3.226	0.204
III/IV	12 (27.27)	7 (16.28)		
Lymph node metastasis				
Positive	14 (31.82)	27 (62.79)	9.718	0.001*
Negative	30 (68.18)	16 (37.21)		
Nodular goiter				
Positive	25 (56.82)	19 (44.19)	3.863	0.077
Negative	19 (43.18)	24 (55.81)		
Multifocality				
Positive	13 (29.55)	14 (33.56)	0.034	1.000
Negative	31 (70.45)	29 (67.44)		
Hashimoto thyroiditis				
Positive	7 (15.91)	8 (18.60)	0.091	0.804
Negative	37 (84.09)	35 (81.40)		

* P < 0 05, Chi squared test P value

### Correlation between lncRNA BLACAT1 and clinicopathological traits of patients with papillary thyroid microcarcinoma (PTMC)

Furthermore, correlations of *BLACAT1* and clinicopathological traits of PTMC patients (n = 62, tumor volume < 1 cm^3^) were also analyzed. We found significant correlations between BLACAT1 expression and LNM and gender (*P *< 0.05, Table 3), but no correlation was found between BLACAT1 and other clinicopathological traits.

**Table 3:** Correlation between lncRNA BLACAT1 and clinicopathological traits in all patients with PTMC

Parameters	lncRNA BLACAT1		χ^2^	P
	Low (%)	High (%)		
	(n = 31)	(n = 31)		
Sex				
Male	11 (35.48)	6 (19.35)	5.823	0.017*
Female	20 (64.52)	25 (80.65)		
Age (years)				
< 45	12 (38.71)	14 (45.16)	0.000	1.000
≥ 45	19 (61.29)	17 (54.84)		
Extrathyroidal extension				
Positive	9 (29.03)	16 (51.61)	2.013	0.364
Negative	22 (70.97)	15 (48.39)		
TNM staging				
I/II	5 (16.13)	4 (12.90)	0.516	0.849
III/IV	26 (83.87)	27 (87.10)		
Lymph node metastasis				
Positive	20 (64.52)	10 (32.26)	9.014	0.005*
Negative	11 (35.48)	21 (67.74)		
Multifocality				
Positive	12 (38.71)	10 (32.26)	0.323	0.9240
Negative	19 (61.29)	21 (67.74)		
Hashimoto thyroiditis				
Positive	8 (25.81)	9 (29.03)	0.092	1.000
Negative	23 (74.19)	22 (70.97)		
Nodular goiter				
Positive	16 (51.61)	13 (41.94)	0.841	0.603
Negative	15 (48.39)	18 (58.06)		

* P < 0 05, Chi squared test P value

### Relationship between lncRNA BLACAT1 and LNM

**Fig. 2:**
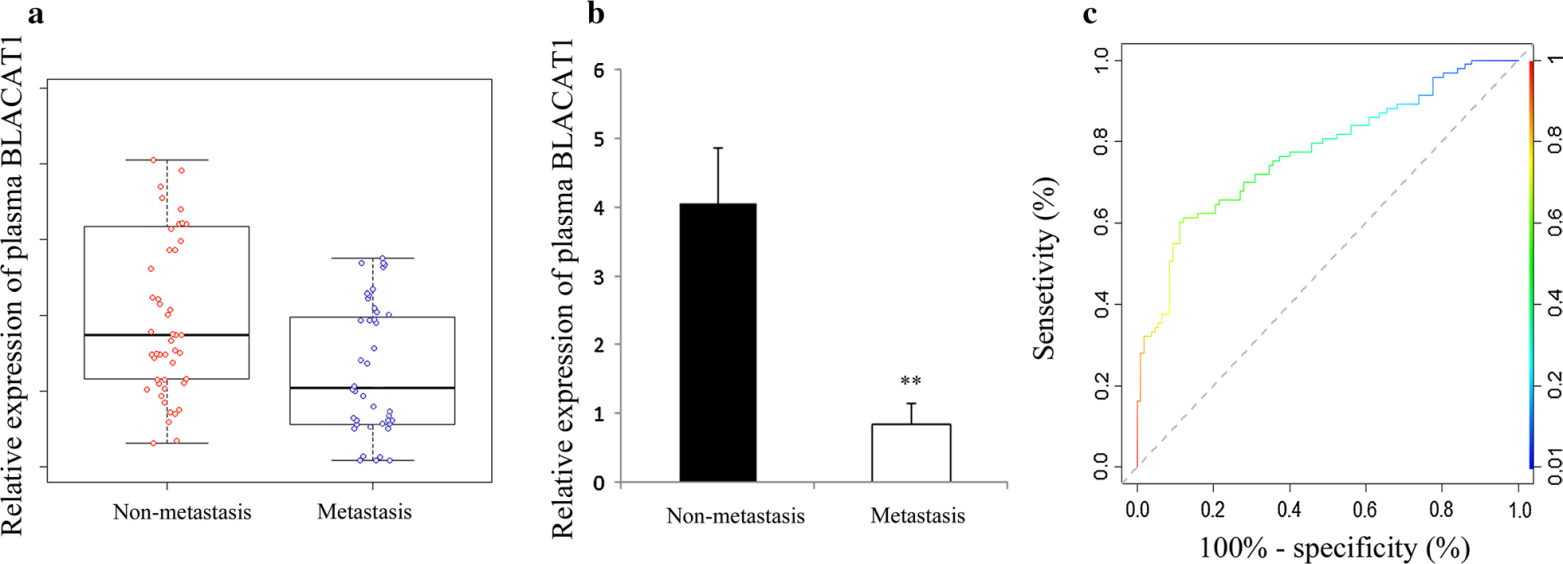
RT-qRCR and ROC curve analysis of BLACAT1 as a diagnostic marker for lymph node metastasis. Scatter plot (**a**) and histogram (**b**) of expression of plasma BLACAT1 in patients with metastasis (n = 41) and non-metastasis (n = 46); **c** ROC curve for evaluating the diagnostic value of BLACAT1 to distinguish PTC patients with metastasis and without metastasis

Expressions of plasma lncRNA BLACAT1 in PTC patients with LNM and without metastasis were compared. Our data showed that PTC patients with LNM had lower plasma BLACAT1 expression (*P *= 0.002, Fig. 2). Multivariable analysis showed that down-regulation of BLACAT1 in plasma was associated with LNM (Table 1). The predicting value of BLACAT1 on LNM was evaluated by ROC curve. When the cutoff value was 1.57, the AUC was 0.825 (95% CI 0.608–0.827, P < 0.001, sensitivity was 77.48%, and specificity was 91.06%) (Fig. 2).

## Discussion

PTC usually shows an excellent prognosis after treatment [14]. In recent years, the rapidly rising incidence of PTC has made it a public health problem. With the application of high throughput sequencing technology, more and more studies have begun to focus on the molecular mechanisms of tumors [15]. Although most patients with PTC have a good prognosis after surgical resection combined with radioiodine and levothyroxine treatment, there are still some patients with metastasis and recurrence [16]. The inadequacy of specific diagnostic markers and treatment strategies is the main cause of death for cancer patients [17]. Therefore, to improve the prevention and treatment of PTC, exploration of molecular mechanisms and identification of new diagnostic and prognostic markers are required.

LncRNAs are non-coding RNAs with more than 200 nucleotides in length, which have various biological functions, including cell proliferation, differentiation, and apoptosis [18, 19]. More and more evidence has demonstrated that lncRNA plays an important role in a variety of cancers [20]. For example, it has been reported that lncRNA BANCR expression is up-regulated in endometrial cancer and it promotes cancer cell proliferation and tumorgenesis [21]. Lnc-GNAT1-1 expression significantly down-regulated in colorectal cancer and it acts as a tumor suppressor through regulating RKIP-NF-κB-Snail circuit [22]. In breast cancer, LncRNA ATB regulates ZEB1 and ZNF-217, inducing epithelial mesenchymal transition, which leads to trastuzumab resistance and increased invasion and metastasis [22]. Another study has found that lncRNA MALAT1 promotes the invasion of gastric cancer cells via binding to the core protein complex PRC2 and inhibiting PCDH10 [23]. Moreover, many studies have been suggested that blood lncRNA could be the potential diagnostic markers for tumors [24, 25]. It has been shown that the expression of lncRNA H19 was abnormal in the blood of patients with gastric cancer [26]. A previous study has been reported that up-regulation of lncRNA BLACAT1 expression in bladder cancer, gastric cancer and colorectal cancer is a poor prognostic factor [13]. However, the plasma expression of lncRNA BLACAT1 in PTC patients and its diagnostic and prognostic values have not been completely elucidated.

In this study, we found that the plasma expression of lncRNA BLACAT1 was significantly lower in PTC patients than that in patients with nodular goiter. We also found that plasma BLACAT1 expression was lower in patients with LNM compared with patients without LNM, whereas there was no difference in BLACAT1 expression among patients with different stages. Similar results were also found in papillary thyroid microcarcinoma. Multivariate analysis indicated that lncRNA BLACAT1 was an independent risk factor of LNM, and the area under the ROC curve of BLACAT1 to distinguish LNM and non-LNM was 0.746. These results suggested that BLACAT1 can be used as a predicting marker of lymph node metastasis. In PTC patients, LNM can be determined by preoperative imaging or intraoperative exploration. LncRNA BLACAT1 in plasma is a potential marker for decision making.

## Conclusion

In conclusion, our results indicated that downregulated plasma BLACAT1 is a potential biomarker for PTC detection, and may predict tumor aggression in patients with PTC.
